# Overview of Three Proliferation Pathways (Wnt, Notch, and Hippo) in Intestine and Immune System and Their Role in Inflammatory Bowel Diseases (IBDs)

**DOI:** 10.3389/fmed.2022.865131

**Published:** 2022-05-23

**Authors:** Seyed Mobin Khoramjoo, Nesa Kazemifard, Shaghayegh Baradaran Ghavami, Maryam Farmani, Shabnam Shahrokh, Hamid Asadzadeh Aghdaei, Ghazal Sherkat, Mohammad Reza Zali

**Affiliations:** ^1^Basic and Molecular Epidemiology of Gastrointestinal Disorders Research Center, Research Institute for Gastroenterology and Liver Diseases, Shahid Beheshti University of Medical Sciences, Tehran, Iran; ^2^Gastroenterology and Liver Diseases Research Center, Research Institute for Gastroenterology and Liver Diseases, Shahid Beheshti University of Medical Sciences, Tehran, Iran; ^3^Faculty of Mashhad Branch, Islamic Azad University, Mashhad, Iran

**Keywords:** inflammatory bowel disease, Wnt signaling, Notch signaling, Hippo signaling, immune system

## Abstract

Inflammatory bowel disease (IBD) is a disorder, which involves the gastrointestinal (GI) tract consisting Crohn's disease (CD) and ulcerative colitis (UC). The etiology of this disease is not yet clear and, hence, there are numerous medications and treatments for patients with IBD, although a definite and permanent treatment is still missing. Therefore, finding novel therapeutic approaches are vital for curing patients with IBD. In the GI tract, there are various lineages of cells with different roles that their existence is necessary for the barrier function of intestinal epithelial cells (IECs). Therefore, signaling pathways, which manage the hemostasis of cell lineages in intestine, such as Wnt, Notch, and Hippo, could have crucial roles in regulation of barrier function in the intestine. Additionally, these signaling pathways function as a governor of cell growth, tissue homeostasis, and organ size. In patients with IBD, recent studies have revealed that these signaling pathways are dysregulated that it could result in depletion or excess of a cell lineage in the intestine. Moreover, dysregulation of these signaling pathways in different cell lineages of the immune system could lead to dysregulation of the immune system's responses in IBD. In this article, we summarized the components and signaling of Wnt, Notch, and Hippo pathways and their role in the intestine and immune system. Furthermore, we reviewed latest scientific literature on the crosstalk among these three signaling pathways in IBD. An overview of these three signaling pathways and their interactions in IBD could provide a novel insight for prospective study directions into finding efficient medications or treatments.

## Introduction

Inflammatory bowel disease (IBD) is a regressive inflammatory condition, which occurs in the gastrointestinal tract (1). Patients with IBD fall into two clinical types: ulcerative colitis (UC) and Crohn's disease (CD). In patients with UC, part of involvement is limited to the colon and it can spread from the rectum to the cecum. In this type of IBD, these parts show large mucosal ulceration. On the other hand, in patients with CD, the parts, which are affected the most, are the ileum and the colon, but other parts of the gastrointestinal (GI) tract could be influenced patchily (2). In spite of the vast studies to find a causative factor for etiology of IBD, it is still known as a multifactorial disorder (3). Different factors, such as internal triggers (genetic susceptibility and immunoregulatory impairments), environmental factors (diet and chemicals), and microbial exposure, are considered to cause IBD (2, 4, 5). Moreover, recent studies have shown that the dysbiosis of gut microbiota profoundly contributes to the development of IBD (2, 6–8).

Various cell lineages arise by differentiation and proliferation of intestinal stem cells (ISCs), which are controlled by multiple signaling pathways, including Hippo, Notch, and Wnt (9–13). These signaling pathways accelerate undifferentiated columnar cells, named crypt base columnar cells, to regenerate into absorptive and secretory cell types in the GI tract (14). Some studies have illustrated that in patients who suffer from IBD, especially those with ulcerative colitis (UC), overexpression of Notch and inhibition of Wnt lead to a lack of Paneth cells that exist in crypts (15). Similarly, other studies have demonstrated that an imbalance in components of Hippo signaling pathway in the intestine of patients with IBD resulted in excess of ISCs and shortage of secretory cells, such as goblet cells and Paneth cells (16). Therefore, dysregulation in pathways that play a role in proliferation and differentiation may explain the defective mucus secretion and wound healing, which could ultimately induce the failure of intestinal barrier in patients with IBD (15). Furthermore, recent studies on Wnt, Notch, and Hippo showed that these signaling pathways play a regulatory role in the function and generation of various immune cells' types that in IBD, it could be dysregulated (17–19). However, numerous studies have been conducted on understanding the function and regulation of the proliferation pathways in the gut epithelium and their specific role in IBDs is still unknown (20).

In IBD, the process of wound healing and mucus secretion is dysregulated that could lead to impaired barrier function of the GI tract and ultimately leaky gut. Herein, we briefly explain the barrier functionality of intestinal epithelial cells (IECs) and the process of wound healing in IBD. In addition, it is illustrated that some proliferation pathways, including Wnt, Notch, and Hippo, could have critical impacts on these processes and the immune system. We also summarize these three signaling pathways and their role in the intestine and immune system. Finally, we concisely discuss the interactions of these signaling pathways in IBD.

## Barrier Function of Intestinal Epithelial Cells

The intestinal epithelial cells (IECs) establish a barrier, which is selectively permeable and sets apart luminal content from beneath tissues (21, 22). Basically, IECs function as a barrier, which prevents unacceptable solutes, microorganisms, viruses, and luminal antigens from passing the epithelium and entering the lamina propria (22, 23). Multiple components that participate in the intestinal barrier consist of the epithelial cells with tight junctions, adherens junctions, and luminal secretions, such as mucus or unstirred layers, on the apical side of the epithelium (22).

## Process of Wound and Mucosal Healing in Inflammatory Bowel Disease

The process of wound healing starts when a part of the intestinal epithelium gets injured. Intestinal wound healing depends on the accurate balance between migration, proliferation, and differentiation of the epithelial cells, which are nearby the wounded area (24). First, epithelial cells surround the wounded area, which loses their columnar polarity. Then, they proliferate to surge the pool of cells for resurfacing the wound. Finally, to maintain the mucosal barrier function, maturation and differentiation of epithelial cells are vital (25). Dysfunction of these three steps during the wound healing's process in patients with IBD results in the broken differentiation and proliferation of different cell lineages in gut, such as goblet cells or Paneth cells, that lead to flawed mucosal secretion and leaky gut (26).

## Importance of Proliferation Pathways in Intestine

Many cell lineages are vital for maintenance of intestinal epithelial barrier integrity, which arise by differentiation and proliferation of intestinal stem cells (ISCs) (27). Crypt-based stem cells, which are near the base of the crypts, need to actively proliferate to maintain continuous renewal of different cell lineages (28). As these cells move up from crypt to villus, proliferation ends gradually and differentiation into one of the four primary cell types occurs (i.e., enterocytes, goblet, Paneth, and enteroendocrine cells) (29, 30). Multiple signaling pathways, such as Hippo, Notch, and Wnt, are responsible for regulating the proliferation and the differentiation in intestinal epithelial cells (9–13, 31). Finally, an imbalance among these types of pathways in epithelium could lead to colorectal cancer and IBD (27).

## WNT Pathway

The Wnt signaling pathways are a group of signaling pathways, which commence with proteins that transmit signals into a cell by cell surface receptors (32, 33). Therefore, this pathway is activated by the cell–cell communications and it has been conserved throughout the biological evolution (27). Wnt pathway is divided into β-catenin dependent (canonical) and β-catenin independent (noncanonical) types (34). This signaling pathway gets activated when Wnt proteins contact with Frizzled (Frz) receptor on the cells' surface (18, 33). Thereafter, Wnt proteins run a complex signaling cascade that plays an important role in regulating cell proliferation and differentiation by regulating the β-catenin, which is an important mediator (34–36). When the Wnt pathway is silenced, β-catenin can be phosphorylated by the ubiquitin-proteasome system [including glycogen synthase kinase 3 (GSK3), casein kinase Iα (CKIα), axin, and adenomatosis polyposis coli (APC)] and then transcription complexes prohibit gene transcriptional activity (37, 38). In opposition, when the Wnt signaling pathway has been activated, β-catenin degradation is banned, which is leading to its aggregation (38, 39). As a result, transcription complexes are changed by accumulated β-catenin, which activates targeted expression of genes related to cell proliferation and migration, EphB2/B3, Cylind-1, and c-Myc (40, 41).

## Role of WNT Pathway In Intestine

Wnt signaling pathway plays a vital role in the intestinal epithelium, specifically in regulating the stem cells' behavior, proliferation, differentiation, and migration (42). This pathway is one of the many signaling pathways for the maintenance of stem cells (43). Recent studies have shown that deletion of β-catenin's encoding gene (CTNNB1) results in disruption of secretory cells' differentiation (44). Wnt pathway is able to direct the beginning development of secretory cells lineage and the endpoint of differentiation of Paneth cells for sustaining homeostasis (45). Reduced Wnt pathway, particularly diminished expression in its transcription factor 4 (TCF-4), could mediate Paneth cells differentiation flaws that it induces specific deficiency of Paneth cell defensins, which is a principal factor in IBD pathogenesis (15, 46). In addition, evidence found in TCF-4 knockout mice illustrated that the reduced level of defensins in gut permits bacteria to invade the epithelium and resulting in colitis (42, 47). On the other hand, excessive Wnt pathway accumulates β-catenin in the cytoplasm, then they are translocated to the nuclear, and finally induces overexpression of Wnt target genes, which lead to colon cancer (48). Furthermore, recent studies on IBD-associated colorectal cancer (CRC) revealed that negative regulators of Wnt, such as AXIN2 and RFN43, are downregulated in 31 tissue sample of patients with IBD-CRC (49).

## Role of WNT Pathway in Immune System

One of the important roles of Wnt pathway is in multiple layers of immune regulation. Presence or absence of Wnt proteins could have impact on different immune cells, such as dendritic cells, macrophages, CD8+ T cells, and CD4+ T cells (18, 50). Based on recent studies on Wnt proteins and dendritic cells (DCs), it is demonstrated that Wnt proteins may be involved in promoting DCs into a tolerogenic state (51). Manicassamy et al. showed that reduced expression of β-catenin in DCs enhances inflammatory responses in the mice model of inflammatory bowel disease. Therefore, in DCs, β-catenin signaling causes a tolerogenic state and prevents them from inflammatory responses (52). Recent studies on Wnt pathway and macrophages showed that in macrophages, Wnt ligands have crucial roles in repairing injured tissues, since macrophages' role in tissue repairing and wound healing is well known (53, 54); however, in some studies, it is demonstrated that macrophage-derived Wnt5a maintains immune functions and stimulates the secretion of proinflammatory cytokines (55). Some previous studies showed that proteins of Wnt signaling play a regulatory role in the function of CD8+ T-cell effector and generating memory T-cell pool (18, 56). Functional regulation of β-catenin-mediated CD8+ T-cell immune responses remains unclear (57–60). The role of Wnt pathway in CD4+ T-cells is not yet clear and it needs more accurate investigations for various Wnt ligands; however, some studies suggested that the overexpression of β-catenin in regulatory T (Treg) cells enhanced Treg function in IBD (61, 62). The canonical Wnt signaling proteins are able to induce their roles in T-cell differentiation and effector function in various inflammatory diseases, such as IBD, cancer, as well as in autoimmunity and viral infections (18). Wnt signaling is important in inflammatory and fibrotic diseases and it is in harmony with the roles of Wnt proteins in repairing injured tissue. Recent studies' outcomes demonstrated that Wnt signaling plays vital roles in lymphomyelopoiesis and immune responses (56). Moreover, Wnt pathway and inflammatory signaling pathways, such as nuclear factor-kappa B (NF-κB), Janus kinase-signal transducer and activator of transcription 3 (JAK-STAT3), and mitogen-activated protein kinase (MAPK), affect each other, which regulate inflammatory factors' secretion during the pathogenesis of colitis (18, 63).

## Notch Pathway

The Notch signaling pathway is conserved during the evolution that is presented in most animals (27). It regulates the differentiation and development of cells, tissues, and organs by interactions among nearby cells (27). The pathway consists of receptors, ligands, transformation complexes, and several regulatory molecules (64). The Notch transmembrane receptor plays a critical role in the signaling pathway that regulates the fate and development of a wide range of metazoan cells through local cell interactions (65–67). There are at least four different Notch receptors (Notch 1–4) in mammals that the Notch-1 is dominant in the intestine (66, 68). As a result of binding ligands, slight structural conformations in the membrane around the binding site activate matrix metalloproteinases (MMPs) and γ-secretase (69). With the assistance of activated γ-secretase and a disintegrin and metalloproteinase (ADAM)-family MMPs, Notch intracellular domain (NICD), which is the activated form of Notch receptors, is generated (64, 66, 70). Thereafter, NICD enters the nuclear and by the help of activator transcription complexes, it regulates the *HES* genes to determine the fate of the cell (64, 71–74).

## Role of Notch Pathway in Intestine

In the intestine, Notch is necessary for the survival of ISCs. Notch is also responsible for determining ISCs differentiation into secretory or absorptive lineages (63). High Notch signaling leads to absorptive differentiation, whereas low Notch signaling induces differentiation of secretory cells (75). Abnormal activity of the Notch pathway in IBD induces increased expression of the HES1 transcription factor in human colon cell lines, which thereafter inhibits differentiation of secretory cell lineages and weakens the mucus barrier, which is linked to chronic colitis (76). Accordingly, it is demonstrated that the Notch signaling pathway has a crucial role in maintaining goblet cells in the lesion of patients with UC that is so important in mucosal and wound healing in IBD. Zheng et al. illustrated that abnormality in expression of Notch intracellular domain (NICD) in ulcers induces reduction in the quantity of goblet cells in patients with UC (77). NICDs imposed expression leads to a decrease in phenotypic genes for goblet cells located in human IECs (77). In addition, several signaling pathways and cytokines cascade with Notch pathway mediate epithelial regeneration, such as interleukin-22 (IL-22) and tumor necrosis factor-α (TNF-α) (27, 78). Accordingly, Kuno et al. found that messenger RNA (mRNA) expression of OFLM4, intestinal stem cell marker, is upregulated by TNF-α and Notch pathway in patients with IBD (63). Moreover, it is reported that Notch signaling pathway contributes to the maintenance of tight junctions and adherens junction proteins in mice. Ahmed et al. showed that during the infection with *Citrobacter rodentium* and absence of Notch pathway, the function of tight junctions and adherens junctions impaired, which could result in increased permeability of epithelial cells and more exposure of luminal contents with immune system and inflammation (79). Notch dysregulation has also been demonstrated in colon cancer (80).

## Role of Notch Pathway in Immune System

Notch signaling is important pervasively all over the immune system, since it has lineage and context-dependent impacts on a broad range of cells. In the immune system, Notch1 ligands, especially Jagged1, are present in regulatory T-cells (Tregs) (17). The activation of Notch1 in dendritic cells (Notch1 intracellular domain) induces the interaction of signaling elements and components that result in overexpression and the transport of pSmad3, which is known to facilitate the effector function of Tregs (17, 81). Notch signaling is also involved in the improvement of inflammatory conditions. Some studies revealed that Notch signaling pathway improves an inflammatory cascade in macrophages in inflammation and blocking Notch reduces the production of proinflammatory cytokines, such as interleukin-1β (IL-1β) (82, 83).

## Hippo Pathway

The Hippo pathway is a pathway, which is remained conserved throughout the evolution and it controls the size and homeostasis of an organ by regulating cell proliferation, survival, apoptosis, and stemness (84, 85). Specifically, intercellular contacts and membrane adhesion complexes modulate the transduction of a signal by the fundamental constituents of this pathway, which are highly conserved in mammals (86). These components contain the mammalian sterile 20-like kinases, MST1 and MST2, with their regulatory protein WW45 (SAV1) and the large tumor suppressor 1 and 2 kinases (LATS1 and LATS2) with their regulatory protein MOBKL1A/B (MOB1) (87). When the MST1/2 kinases get activated and LATS kinases get phosphorylated, it leads to negative regulation of cell proliferation (88). Concisely, phosphorylation of LATS kinases results in a process of phosphorylation of the transcriptional coactivators Yes-associated protein (YAP) at Ser127 and PDZ-binding motif (TAZ) at Ser89, so it makes binding sites for 14-3-3 proteins that accumulate YAP/TAZ in the cytoplasm (88). Once this inhibitory phosphorylation does not work, these transcription factors will be able to enter to the nucleus and contact with other transcriptional factors that enhance cell proliferation (89, 90). In cells that are in apoptotic phase because of exposure to severe DNA damage stress, YAP activates the transcription of proapoptotic genes through binding to the p73 transcription factor that is a p53-like tumor suppressor. This process is moderated by phosphorylation of YAP at the Tyr357 position through c-Abl protein, which provides a higher affinity of YAP compared to p73 (89, 91).

## Role of Hippo Pathway in Intestine

It is confirmed that YAP/TAZ enhances regeneration of tissues in the mammalian intestine (92). Accordingly, Yui et al. demonstrated that YAP/TAZ is associated with the expression of Sca1, which is a cell surface protein representing a marker for the repairing epithelium (93). YAP/TAZ has two different roles in the renewal of the intestinal epithelium: one is ISCs' proliferation that happens through collaboration of YAP/TAZ with transcription factor TEADs (94) and the other role is encouraging goblet cells differentiation by cooperation with transcription factor klf4 (95, 96). The activity of MST1/2 gets higher, as cells move from the crypts toward the lumen, so MST1/2 has decreased activity in the crypts (97). Contrarily, YAP is plentiful in the nucleus of the cells, which are located in lower crypts; however, this molecule is also found in cytoplasm of upper cells in the villi (97). In general, the expression of YAP in the nucleus of cells diminishes as cells move from the crypts to the villi; in contrary, expression of YAP in cytoplasm surges (16). Deletion of MST1/2 in mouse intestinal epithelium cells induces an improved amount of nuclear YAP; as a result, it increases proliferation of undifferentiated ISCs and lack of secretory cells both in the small and large intestines (16). In another study, it is demonstrated that in IECs, conditional knockout of MST1/2 results in disorganized villus structures, increased undifferentiated cells, and dysplastic epithelia (98). It is also reported that in mouse gut, deficiency of SAV1 induces enlargement of crypt structures (92). YAP modulates the regeneration of mucus both in the patients with IBD and the DSS-induced colitis mouse model (99). Moreover, previous studies conducted by Ou et al. illustrated that YAP/TAZ expression is linked with promotion of fibrosis in patients with CD by activating intestinal fibroblasts (100). To sum up, YAP in the nucleus has a positive role in the regeneration of intestinal epithelium in IBD and may provide a novel therapeutic target for IBD.

## Role of Hippo Pathway in Immune System

Recent studies have shown that the Hippo pathway plays an important role in the modulation of immune system. MST1/2 has a pivotal role in mediating, migration, adherence, and survival of T cells by its downstream effectors, such as LATS1/2, NDR1/2, and YAP (101). MST1/2 promotes the function of regulatory T-cell (Treg) through modulating Foxp3 acetylation (16, 102). It is shown the deficiency of MST1/2 leads to impairment of Foxp3 expression and Treg cell development and its function in mice (16, 102). It is also illustrated that the deficiency of MST1/2 might induce the lack of naïve T cells, which could lead to autoimmune demonstrations or resulting in recurrent bacterial or viral infections (103, 104). Additionally, in a study by Geng et al., it has been found that the TAZ is able to determine the fate of a T cell to become a proinflammatory T-helper (Th) 17 cell or an immunosuppressive Treg cell (105). Particularly, lack of TAZ improves the differentiation of Treg cells; however, activation of TAZ enhances Th17 cell differentiation (105). On the other hand, YAP in macrophages was shown to deteriorate the IBD, since it negatively affects M2 polarization of macrophages, which is induced by IL-4/IL-13 and promotes the activation of M1 macrophages that is caused by lipopolysaccharide (LPS) or interferon-γ (IFN-γ) (19).

## Crosstalk Between WNT, Notch, and Hippo Signaling Pathways in Inflammatory Bowel Disease

**Figure 1:**
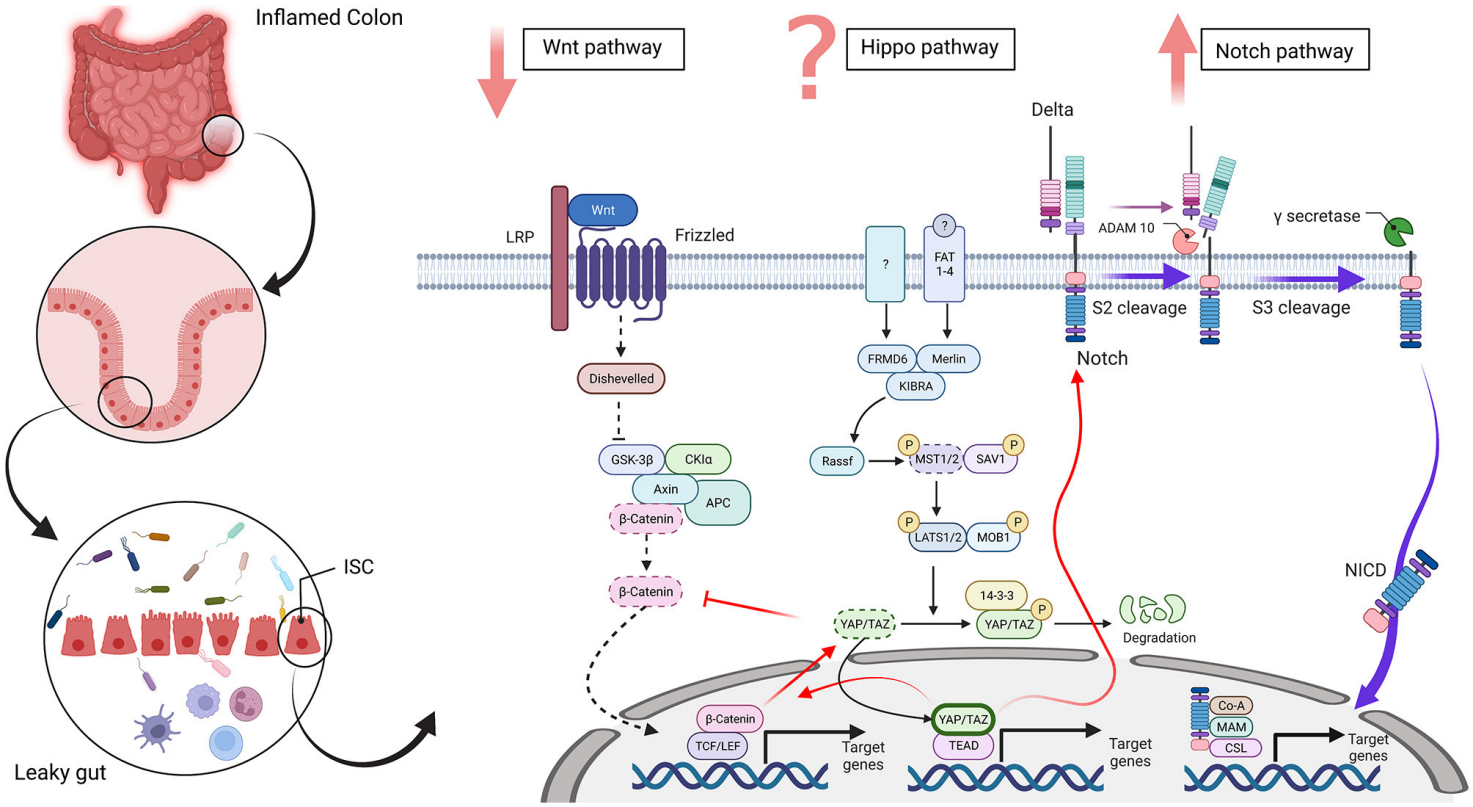
Wnt, Hippo, and Notch signaling in an intestinal stem cell [inflammatory bowel disease (IBD) condition]. The disrupted intestinal epithelial barrier promotes a leaky gut in inflammatory conditions, such as IBD. In this state, differentiation-related pathways, such as Wnt, Hippo, and Notch, are dysregulated in intestinal stem cells. The Wnt signaling pathway plays a vital role in the intestinal epithelium and downregulation of β-catenin disrupts the differentiation of secretory cells in IBD. The Hippo pathway regulates cell proliferation, survival, apoptosis, and stemness. An improved amount of nuclear YAP/TAZ occurred in IBD and increased the proliferation of undifferentiated intestinal stem cells (ISCs) and a lack of secretory cells. In contrast, cytoplasmic YAP/TAZ overexpression is seen in inflamed tissue. Also, MST1/2 is downregulated and disorganized villus structures and increased undifferentiated cells and dysplastic epithelia. However, it is controversial that Hippo pathway is upregulated or downregulated in IBD. The Notch pathway regulates the differentiation and development of cells, tissues, and organs by interactions among nearby cells. Upregulation of the Notch pathway in IBD inhibits differentiation of secretory cell lineages and abnormality in the expression of NICD in ulcers reduces the quantity of goblet cells in patients with ulcerative colitis (UC). Red arrows show crosstalk between these three pathways and dotted lines display downregulated pathways and molecules. Abbreviations: low-density-lipoprotein-related protein (LRP), adenomatosis polyposis coli (APC), glycogen synthase kinase 3 beta (GSK3β), casein kinase 1α (CK1α), T-cell factor (TCF), lymphoid enhancer factor (LEF), FERM domain-containing protein 6 (FRMD6), mammalian Sterile 20-related 1 and 2 kinases (MST1 and MST2), Salvador 1 (SAV1), Large tumor suppressor 1 and 2 kinases (LATS1 and LATS2), Mps One Binder Kinase Activator-Like 1A (MOB1), Yes-associated protein 1 (YAP), PDZ-binding motif (TAZ), TEA domain family member (TEAD), Fat-related atypical cadherins 1-4 (FAT 1-4), A Disintegrin and metalloproteinase domain-containing protein 10 (ADAM 10), Notch intracellular domain (NICD), meprin A-5 protein (MAM) and CBF1, Suppressor of Hairless, Lag-1 (CSL). The figure has drawn by BioRender (www.Biorender.com).

There is an interplay among the proliferation signaling pathways, including Hippo, Wnt, and Notch, in intestinal regeneration (Figure 1) and imbalance among these pathways results in different problems, which are associated with different diseases, including IBD.

Once the Hippo pathway gets activated, it negatively affects the Wnt signaling pathway by cytoplasmic and phosphorylated YAP/TAZ; however, deactivation of the Hippo pathway has a positive effect on the expression of Wnt target genes by nuclear and dephosphorylated YAP (20). Additionally, it is reported that β-catenin activates and upregulates YAP and TAZ (106, 107). Imajo et al. illustrated that the YAP/TAZ regulates Wnt signaling that relies on the state of phosphorylation and cellular localization of YAP/TAZ proteins (108). Cytoplasmic YAP/TAZ downregulates the Wnt signaling through the regulation of nuclear translocation and activation of β-catenin (108, 109). This is in contrast to nuclear YAP, which stabilizes β-catenin and resulting in the improved expression of Wnt target genes (110, 111). It is shown that YAP and β-catenin grow in nucleus within regeneration following inflammation. Once the nuclear YAP is overexpressed, it enhances Wnt/β-catenin signaling and significantly leads to the improvement of the IECs' healing ability, thereby demonstrating that nuclear YAP improves the IECs' proliferation by the activation of Wnt/β-catenin signaling pathways (112). In addition, within intestinal regeneration following tissue damage, cytoplasmic YAP restricts Wnt signals, interrupts the ISCs, and decreases the stem cells' growth, which induce abnormal migration of Paneth cells and reduction of ISCs (97).

In previous studies, it is reported that the Hippo pathway is able to regulate Notch signaling. In intestine, conditional knockout of MST1/2 leads to increased amount of NICD in nucleus (16). Reduction of MST1/2 results in activation of Notch signaling through decreasing of phosphorylation and increasing the abundance of nuclear accumulation of YAP (16). Intrinsically, the YAP molecules, which are located in nucleus, facilitate Notch signaling (98). Moreover, it is shown that administration of gamma-secretase inhibitors (GSIs), which restrict YAP-activating Notch, induce colitis (98, 113). In general, the Hippo pathway is able to downregulate Notch signaling *via* phosphorylation and suppression of the YAP.

Wnt and Notch signaling pathways are so intertwined that it has been suggested that they established an integrated signaling termed as “Wntch” (114). An accurate balance between Wnt and Notch is required for the homeostasis of intestine, as their dysregulation may result in inflammation, colitis, and tumorigenesis. It is demonstrated that activation of Notch pathway upregulates the expression of β-catenin (115). On the other hand, Kay et al., by utilizing chemical reaction network theory (CRNT), found that Wnt-mediated actions on Hes1 promoter are able to change dynamic transition of Notch signaling pathway from multistability to monostability, highlighting the role of β-catenin in modulating Notch signaling pathway (116).

## Discussion

In this short article, we aimed to overlook on three proliferation pathways playing important roles in intestine and immune system. We also reviewed some of their impacts on pathogenesis of IBD. In the process of wound and mucosal healing in a healthy condition, adjacent cells to the lesion start to proliferate and migrate to retrieve the columnar polarity of the epithelial cells (24). This process is faulty in the intestine of patients with IBD, particularly those who have UC. Multiple factors contribute in this flaw, but it is shown that one of the most important factors is the dysregulation of proliferation pathways, such as Hippo, Wnt, and Notch, and also an imbalance among them (27). Moreover, dysregulation of these pathways leads to an imbalance of cell lineages in intestine. Importantly, this dysregulation results in depletion of goblet cells and Paneth cells, which leads to impaired secretion of mucus and defensins and invasion of various bacteria to epithelial cells (26). Accordingly, this results in massive responses of the immune system and inflammation that cause tissue damage and ulcers, which are the clinical symptoms of IBD (117). These signaling pathways also play important roles in various immune cells, including dendritic cells, macrophages, and T cells. These signaling pathways could impact immune cells to differentiate to a particular type that it could attenuate or strengthen immune responses (117). Moreover, there is a crosstalk between these signaling pathways and inflammatory signaling pathways, such as NF-κB, JAK-STAT3, and MAPK, that could influence an immune cell to produce proinflammatory or anti-inflammatory cytokines (104). Dysregulation in these pathways in immune cells is reported to be important in immune responses to inflammation in patients with IBD (117).

## Future Perspective

This study needs more investigation in different aspects. First, a precise analysis of the relationship among these three pathways needs to be more investigated. By doing so, we will be able to understand the impact of different molecules of signaling pathways on each other. In addition, current findings demonstrated the role of these pathways in several immune cells yet not all of them. More experiments could be done in finding their role in other immune cells. Finally, these proliferation pathways can be a potential target for medications. More studies are required to develop efficient drugs for triggering epithelial cells to regulate these pathways. The results of prospective studies can dwindle the morbidity and mortality linked to IBD, hence reduce the worldwide incidence of this disease.

## Author Contributions

SMK: literature search and writing. SBG, MF, SS, HA, GS, and MZ: literature search. NK: drawing of figure. All authors contributed to the article and approved the submitted version.

## Conflict of Interest

The authors declare that the research was conducted in the absence of any commercial or financial relationships that could be construed as a potential conflict of interest.

## Publisher's Note

All claims expressed in this article are solely those of the authors and do not necessarily represent those of their affiliated organizations, or those of the publisher, the editors and the reviewers. Any product that may be evaluated in this article, or claim that may be made by its manufacturer, is not guaranteed or endorsed by the publisher.
